# Photon Energy Dependent Micro-Raman Spectroscopy with a Continuum Laser Source

**DOI:** 10.1038/s41598-018-29921-6

**Published:** 2018-08-02

**Authors:** Stefan Krause, Marc H. Overgaard, Tom Vosch

**Affiliations:** Nano-Science Center/Department of Chemistry, University of Copenhagen, Universitetsparken 5, 2100 Copenhagen, Denmark

## Abstract

We present a method for continuous, photon energy dependent micro Raman spectroscopy. A narrow excitation line is selected from a continuum laser by an acousto-optic tunable filter (AOTF) plus an additional monochromator (MC). Automation of laser, AOTF, MC and tunable long pass filters enables us to continuously scan the wavelength over the full visible range while synchronously acquiring Raman spectra over a photon energy range from 1.85 eV to 2.83 eV. We demonstrate the applicability of our method on a well-studied sample, reduced graphene oxide (rGO), where we measure the Raman scattering over the whole visual range and use the photon energy dependence of the D, G and G^S^ band as verification for the method we present here. We complement this set of data with additional results from a Ti:sapphire laser source, covering the 1.75 to 1.41 eV range. From the full photon energy range of 1.41 to 2.83 eV, we noticed a small deviation from linearity for the dispersion of the D band.

## Introduction

Besides the typical photon energy (~ν^4^) dependence of the Raman signal^1^, many materials^2–4^ as well as nanomaterials, such as quantum dots^5,6^, carbon nanotubes^7–11^, semiconductors^12^, semiconducting nanowires^13,14^ and graphene^15,16^ show resonances at specific photon energy ranges accompanied by strong enhancement of the Raman signal by several orders of magnitude^2,3,9,16–19^. Accurate determination of these resonances requires light sources with large tunable energy ranges and narrow emission lines. Commonly used gas lasers (Ar^+^, Kr^+^, HeNe …) provide only a few distinct emission lines within the visible range not capable of covering photon energies in a quasi-continuous way. Although tunable sources like dye lasers, Ti:sapphire lasers and OPOs are available, they usually require realignment to change the wavelength over a large spectral range. Here we demonstrate a solution by applying a continuum laser source which can provide a continuous emission spectrum typically ranging from 3.1 eV (400 nm) to 0.517 eV (2400 nm). Selection of the desired excitation energy (2.83 to 1.85 eV) for the Raman measurement was here achieved by sending the laser continuum output through an AOTF and an additional monochromator (MC). The power demanding NIR range from 1.75 to 1.41 eV was covered with and additional Ti:sapphire laser which was sent through the monochromator as well. In combination with tunable long pass filters, this approach enables for recording Raman signals in the best case from approximately 200 cm^−1^ while continuously scanning the photon energy. To demonstrate our method, we used rGO, which provides a good Raman signal over the whole visible range^20–22^. The well-characterized Raman bands of rGO in the literature act as a good reference to validate our new method^23,24^. The reduced graphene oxide (rGO) was synthesized according to the method from Eigler *et al*. and recently used as an ink for printing semitransparent electrodes^25,26^.

## Results and Discussion

Besides having sufficient intensity, a Raman spectroscopy light source should offer a spectral narrow emission line. Therefore, gas ion lasers are typically used since the underlying atomic transitions provide very sharp laser lines. Unfortunately, these gas lasers only provide fixed photon energies which prevent continuous scanning of the energy in Raman spectroscopy.

In recent years, continuum laser sources have become available and offer an alternative possibility delivering a continuous spectrum. Implementing AOTFs and MCs enables to select a specific photon energy. This approach delivers laser tuning in steps only limited by the MC (~0.1 nm for 600 grooves/mm grating). Due to typical light scatter levels (10^−4^–10^−6^) in commonly used MCs, suppression of the undesired photon energies might not be sufficient^27^. These residuals can be further suppressed by applying double MCs or simply sending the laser light twice through a single MC as performed in this work. The scanning of the excitation energy can easily be achieved by turning the MC grating which is usually equipped with a stepper motor. The continuous sweep of excitation energy requires also an adaptation of the long pass filters in case of monitoring low wavenumber Raman bands. This adaptation is accomplished by tunable long pass filters whose transmission depends on the incidence angle of the incoming light.

**Figure 1:**
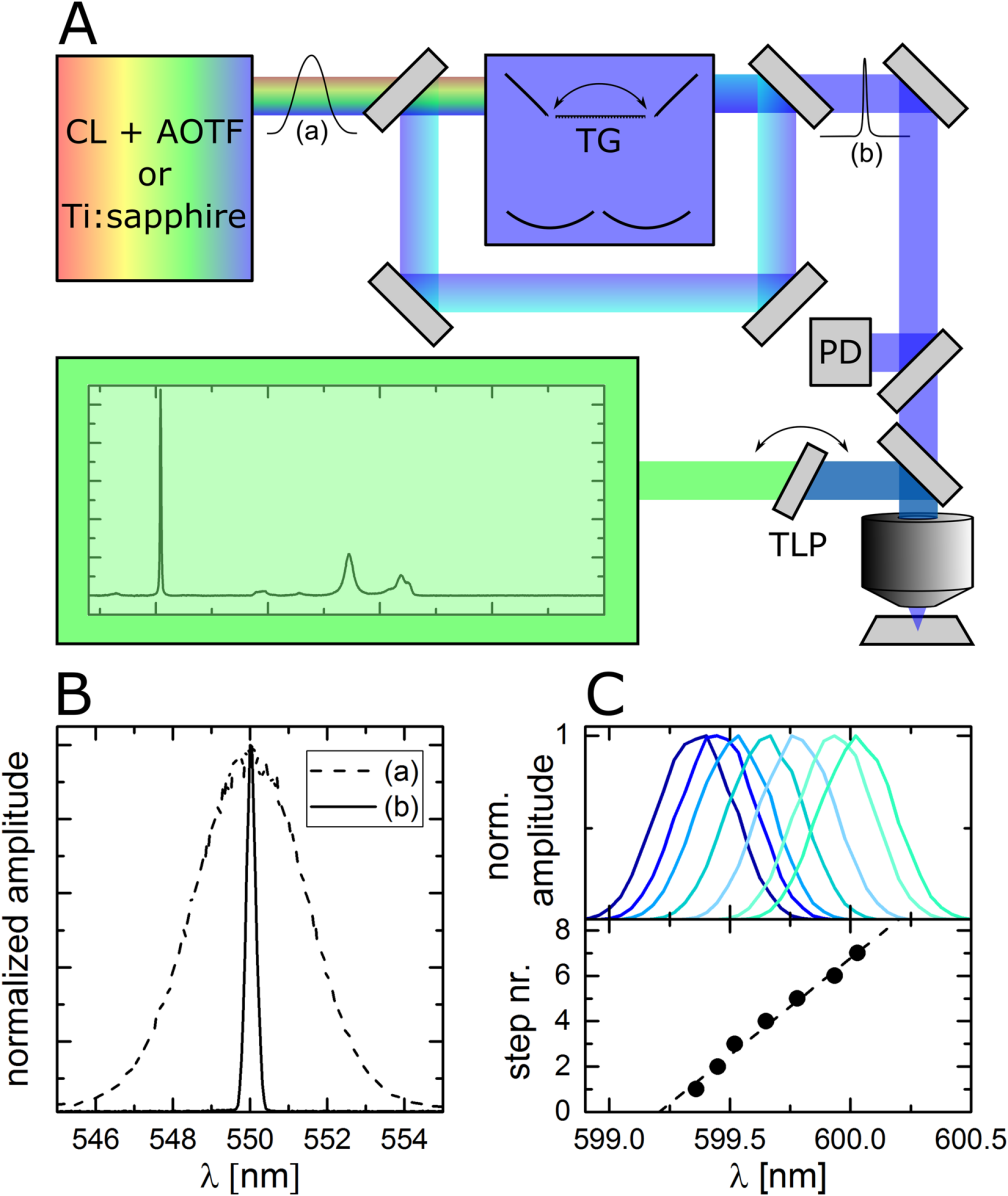
(**A**) Scheme of the experimental setup consisting of a continuum laser (CL) with acousto-optic tunable filter (AOTF) or a Ti:sapphire laser, monochromator with tunable grating (TG), confocal microscope, photodiode (PD), tunable long pass filter (TLP) and spectrometer. (**B**) Measured laser line width without (dashed) and with (solid) monochromator. (**C**) Consecutive laser spectra for the smallest available monochromator step widths and monochromator steps as a function of laser peak positions giving a slope of about 8.5 steps/nm.

In the present experiment, six different tunable filters are used to cover the full visible and parts of the NIR range. Figure 1A shows a schematic representation of the experimental setup. A continuum laser (SuperK EXTREME EXB-6 with SuperK SELECT AOTF wavelength selector) was integrated into a confocal microscope to cover the visible excitation range. The SuperK SELECT provides a laser line of about 3.5 nm full width at half maximum (see Fig. 1B dashed line). The light is sent twice through a Czerny-Turner-type MC (Acton Research SP300i) with a blazed grating of 600 grooves/mm and a focusing length of 0.3 m resulting in an adjustable laser line of 0.3 nm full width at half maximum and a minimum step size of about 0.12 nm (see Fig. 1B solid line). For measuring Raman spectra in the NIR range (1.75 to 1.41 eV) a Ti:sapphire laser is used and sent through the monochromator as well for cleaning up the laser line. The laser light is then reflected on a 30:70 beam splitter (XF122 Omega Optical) and focused onto the sample by an oil immersion objective (Olympus, UPLFLN 100× NA = 1.3). According to the laser output the intensity varies between 0.25 to 0.5 kW/cm^2^ in the visible range for the continuum laser and between 30 to 200 kW/cm^2^ in the NIR range for the Ti:sapphire laser (see supporting information). The Raman signal is collected by the same objective. Afterwards, the remaining laser light is blocked by a tunable long pass filter chosen according to the actual excitation range (VersaChrome Edge tunable long pass filters, 448 nm to 501 nm, 501 nm to 561 nm, 561 nm to 628 nm, 628 nm to 704 nm, 697 nm to 805 nm and 783 nm to 905 nm, Semrock) and mounted on a home-buildt rotational stage while the Raman signal is acquired with a spectrograph (Princeton Instruments SPEC-10:100B/LN_eXcelon CCD camera, SP 2356 spectrometer, 300 or 1200 grooves/mm). Wavelength calibration of all measurement ranges was performed with a neon spectral lamp (6032 Newport). This includes also correction of the tilting influence of the tunable filters. The silicon peak of the substrate at 520 cm^−1^ served as a reference point in all spectra since it is more accurate than the MC determined excitation energy. A 10:90 beam splitter provided the signal for laser power correction which was acquired with a photodiode power sensor (S120VC Thorlabs). Synchronization of laser, monochromator, tunable long pass filters, spectrograph and photometer was accomplished by self-written LabVIEW routines.

As compared to standard Raman instruments with fixed laser sources the clear advantage of our approach is the flexibility in choosing variable excitation energies over a broad range from theoretically 400 nm to 2400 nm. The light source can be tuned in steps of 0.12 nm as mentioned above. The most limiting factor for applications is the comparably low available excitation power (up to 0.5 kW/cm^2^, see Fig. S1 supporting information) which requires long exposure times of up to several 100 seconds. Measuring higher resolution Raman spectra (for example with 1200 grooves/mm gratings and above) will be even more time consuming, since the signal of a Raman band will be spread over more pixels. However, searching for resonances in materials like CNTs should be facilitated by the versatility of the proposed method. In addition, one must be aware that a tunable laser source always requires either a separate precise determination of the current photon energy or a reference Raman signal, which in our case is given by the silicon peak.

We demonstrate the feasibility of our Raman scanning approach on a sample of rGO synthesized according to the method from Eigler *et al*.^25^. rGO as well as graphite and graphene show strong Raman signals making these samples ideal for our demonstration. Particularly suitable are the D and G bands, which arise from bond stretching of pairs of sp^2^ atoms and the breathing modes of sp^2^ atoms^21,22^. The sample was prepared by oxidizing graphite flakes in concentrated sulfuric acid and permanganate under careful cooling. Subsequently, the resulting graphene oxide was spin coated onto a silicon wafer and reduced with a 1:1 mixture of trifluoroacetic acid and hydroiodic acid. The vapor generated the reduced graphene oxide^26^.

**Figure 2:**
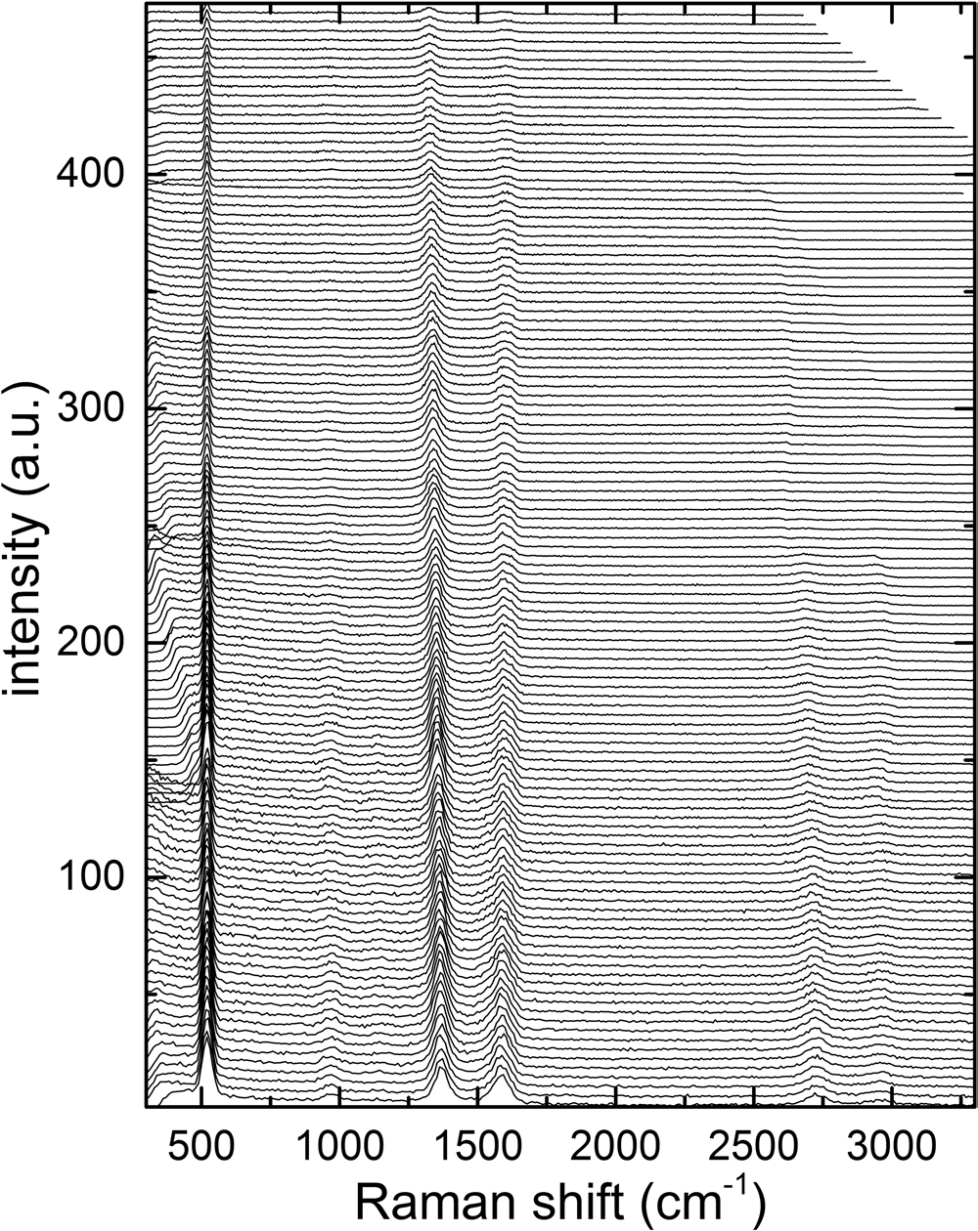
Raman spectra of rGO in the energy range from 2.83 eV (bottom) to 1.85 eV (top).

Raman spectra for visible excitation light were recorded for 150 s in the range from 2.83 eV to 2.20 eV and for 200 s in the range from 2.20 eV to 1.85 eV. The spectra are shown in Fig. 2. Additional Raman spectra for NIR excitation from 1.75 eV to 1.41 eV can be found in Fig. S2 in the supporting information. The assignment of the bands was done based on the nomenclature by Heller *et al*.^28^. The shoulder of the G peak, becoming more pronounced at excitation energies below 2.5 eV, is historically and more commonly referred to as D’, but we will use in this manuscript G^S^ instead^22,29–31^. Besides the pronounced peak of the silicon substrate at 520 cm^−1^ the D and G band are present at about 1370 cm^−1^ and 1590 cm^−1^ for the highest photon energy^16,22^. At this energy, the D feature has an amplitude about twice as much as the G feature. In addition, the 2D and D + G^S^ are visible at 2695 cm^−1^ and 2935 cm^−1^, respectively. Depending on the transmission profile of the tunable long pass filters that we used, a maximum transmission window of approximately 200 cm^−1^ to 3700 cm^−1^ was achieved for specific photon energies, while a minimum transmission range of 500 cm^−1^ to 2300 cm^−1^ was achieved over the whole 2.83 eV - 1.85 eV energy range. The latter can be seen for example as some of the higher wavenumber 2D and D + G^S^ bands are cut-off at specific energies.

**Figure 3:**
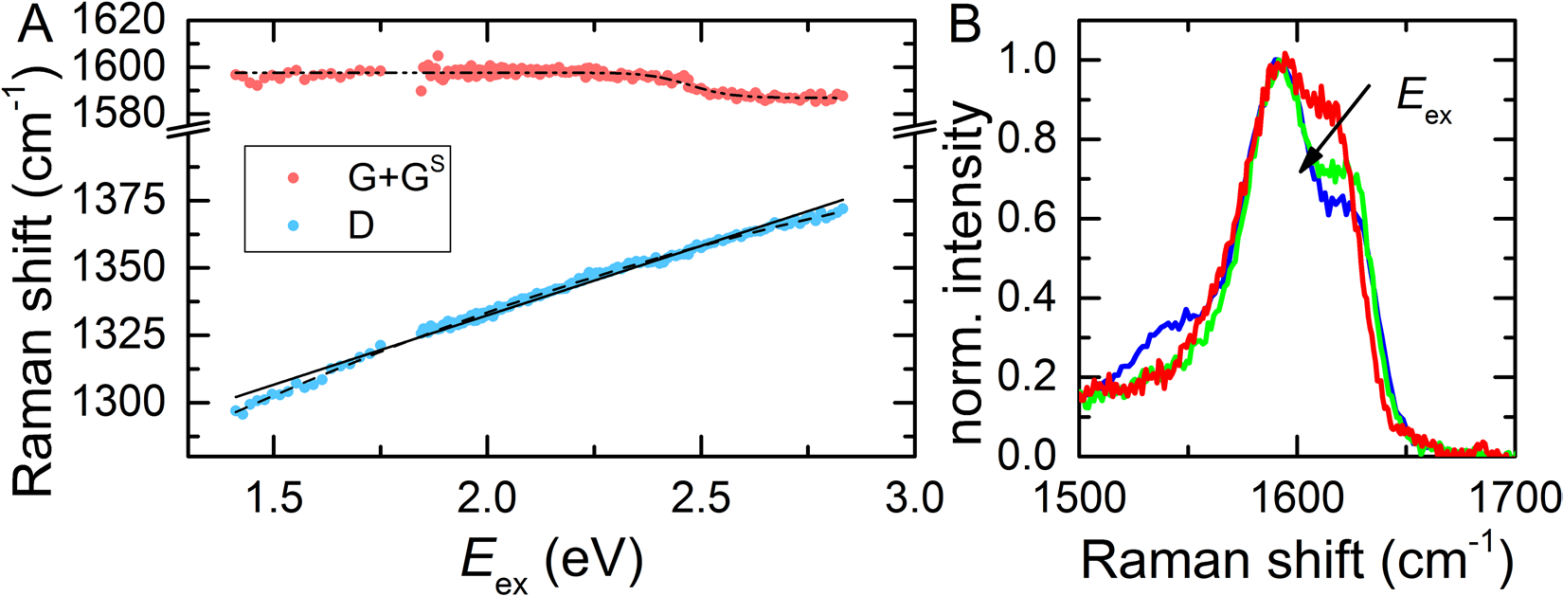
(**A**) Dispersion of D band and the two overlapping bands G and G^S^ as a function of excitation energy. The black dash-dotted line serves as a guide to the eye. The black solid line represents a linear fit to the D band dispersion. The black dashed line represents a parabolic fit to the D band dispersion. (**B**) Three representative high-resolution sections of the Raman spectra for different photon energies (blue: 2.54 eV, green: 2.28 eV, red: 1.96 eV) showing the overlapping G and G^S^ band. The spectra were normalized to the maximum value of the G band. The amplitude of G^S^ decreases with increasing photon energy.

As previously reported, the position of the disorder-induced D band shows a photon energy dependence with a shift of about 50 cm^−1^/eV^21,22,32–34^. We applied Lorentzian fits to the D and G peak features to determine the peak positions and compare them to the literature. The peak positions are shown in Fig. 3A as a function of photon energy. In agreement with previous studies on carbon materials, the D band features a shift from 1374 ± 5 cm^−1^ at 2.83 eV to 1297 ± 5 cm^−1^ at 1.41 eV^21,32–34^. Based on a linear fit to the data, we obtained a shift of (51.5 ± 0.5) cm^−1^/eV, in agreement with the literature^33^. However, precise inspection of the D-related data in Fig. 3A show a systematic deviaton from a linear energy dependency which can be best fitted by a parabolic function. A similar trend can be seen in the data from Pócsik *et al*. which, however, has not been further discussed^32^. In addition, the G band is not expected to show a significant dispersion as was demonstrated for microcrystalline graphite and in the framework of Kramers-Heisenberg-Dirac theory based calculations^28,32^. Nevertheless, as shown here in Fig. 3A and in other studies, the G feature seems to shift with decreasing photon energy from 1585 ± 5 cm^−1^ at 2.83 eV to about 1600 ± 5 cm^−1^ at 1.85 eV^35,36^. We interpret this apparent shift as a consequence of the overlap between the G band and the defect induced G^S^ band with increased amplitudes of G^S^ at lower photon energies^28,37–41^. This causes an apparent shift of the Raman frequencies when fitting the spectra with only one Lorentzian peak. This is also consistent with findings from King *et al*.^36^. To verify this, we acquired spectra with higher resolution (spectrometer grating with 1200 grooves/mm) and by using more intense gas lasers (75–125 kW/cm^2^, approximately 100 times higher intensity than the CL). Three representative spectra for 2.54 eV (blue), 2.28 eV (green) and 1.96 eV (red) are shown in the range between 1500 cm^−1^ and 1700 cm^−1^ in Fig. 3B). From the spectra, it is obvious that G and G^S^ are overlapping, which results in an apparent photon energy dependent shift when fitting with only one Lorentzian peak^35,38^.

## Conclusion

We introduced a novel approach of continuous scanning Raman spectroscopy based on a continuum laser source which enables Raman spectroscopy over the whole visible (and essentially also NIR) range. Applying our method to chemically reduced graphene oxide^25,26^, we acquired consecutive Raman spectra excited over the full visible range (438–672 nm) and parts of the NIR range (710–880 nm). Analysis of Raman spectra by fitting the D and G band with single Lorentzian functions revealed good literature agreement with the observed energy dispersions of both bands. The shift of about (51.5 ± 0.5) cm^−1^/eV for the D band is in good agreement with literature. However, a so far not so often discussed minor deviation from a linear dispersion is observed. Furthermore, we can attribute the apparent shift of the G band to an overlap of G and G^S^ ^21,32^. For the presented method, the spectral resolution with respect to the laser linewidth and step size, is determined by the utilized diffraction grating of the monochromator and can be adapted to the specific material under investigation. Finally, more powerful continuum laser sources than the one we used, will result in a reduced integration time per spectrum. Given the application of Raman spectroscopy in all fields of materials sciences, we believe that our approach can add versatility and easy access to photon energy dependent Raman spectra.

## Electronic supplementary material

Supporting Information
