# Supplementary material for: Photon Energy Dependent Micro-Raman Spectroscopy with a Continuum Laser Source
Source: Sci Rep. 2018 Aug 2;8:11621. doi: 10.1038/s41598-018-29921-6 (PMC6072736; doi:10.1038/s41598-018-29921-6)
Supplement: Supplementary file 1 — Supporting Information [file 41598_2018_29921_MOESM1_ESM.docx]

Supporting Information to

Photon Energy Dependent Micro-Raman Spectroscopy with a Continuum Laser Source.

Stefan Krause*, Marc H. Overgaard and Tom Vosch

*Nano-Science Center / Department of Chemistry*

*University of Copenhagen*

*Universitetsparken 5, 2100 Copenhagen, Denmark*


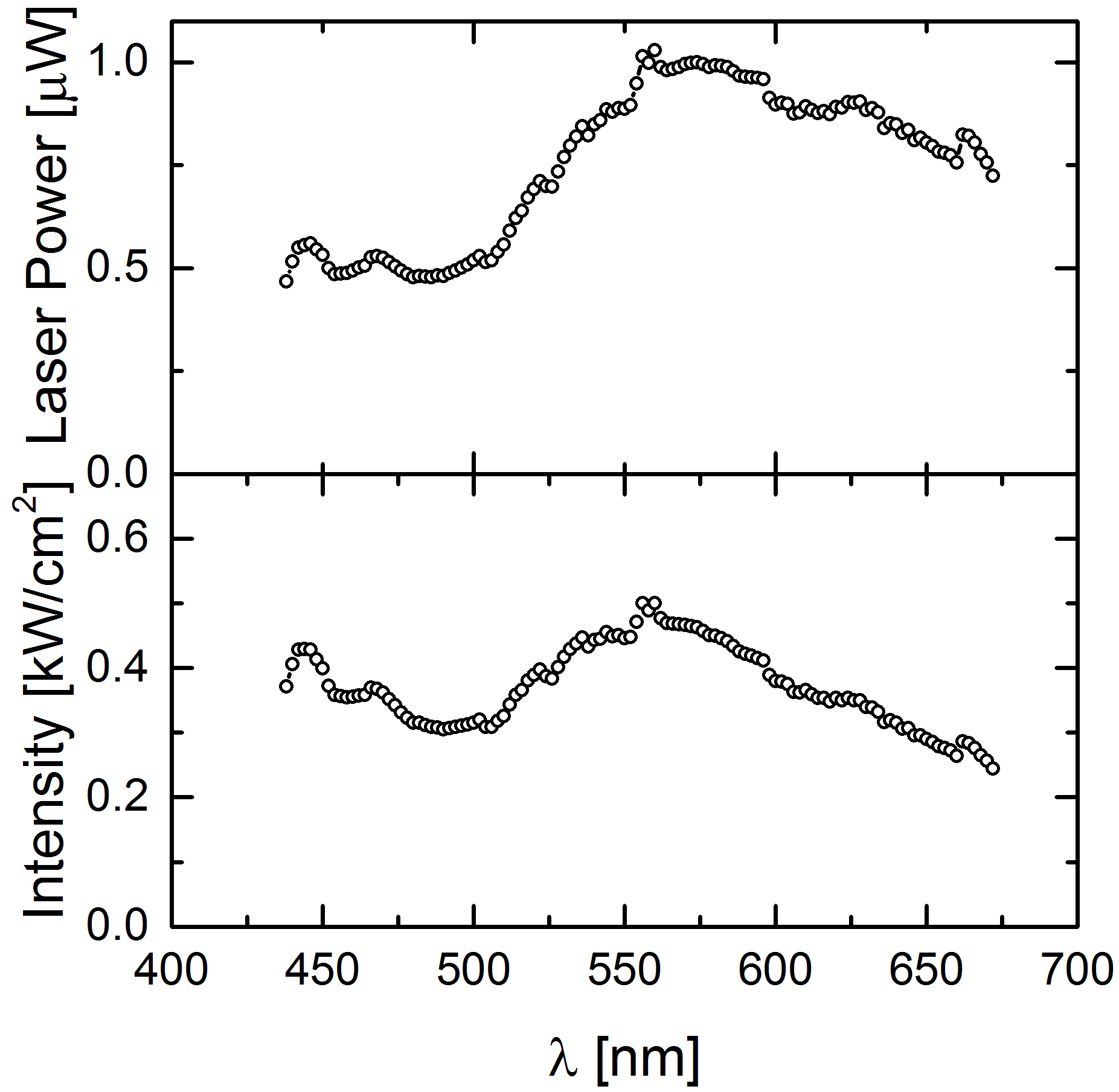


Figure S1: Top) Laser power of the continuum white light laser as a function of excitation wavelength. The power was obtained by measuring 10% of the monochromator output with a photo diode. Bottom) Laser intensity as obtained from the laser power by taking into account the 1/e^2^ – area of the point spread function of the focused laser spot and losses due to beam splitter and objective.


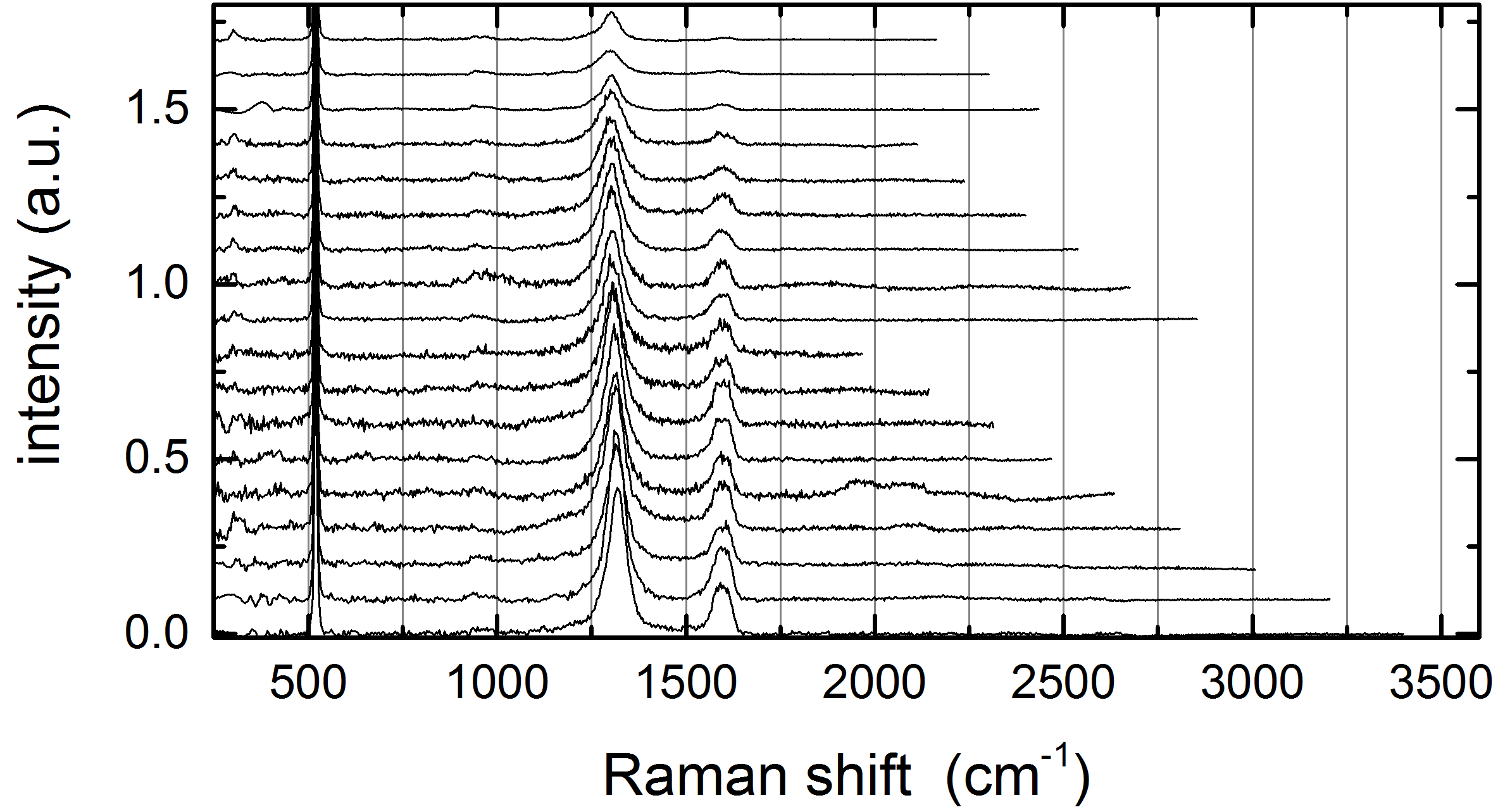


Figure S2: *Raman spectra of rGO in the energy range from 1.75 eV (equals 710 nm, bottom) to 1.41 eV (equals 880 nm, top). Integration time was 60 s for the range 710 to 790 nm and for the range 830 to 850 nm, 30 s for the range 800 to 810 nm and 200 s for the range from 860 to 880 nm.*


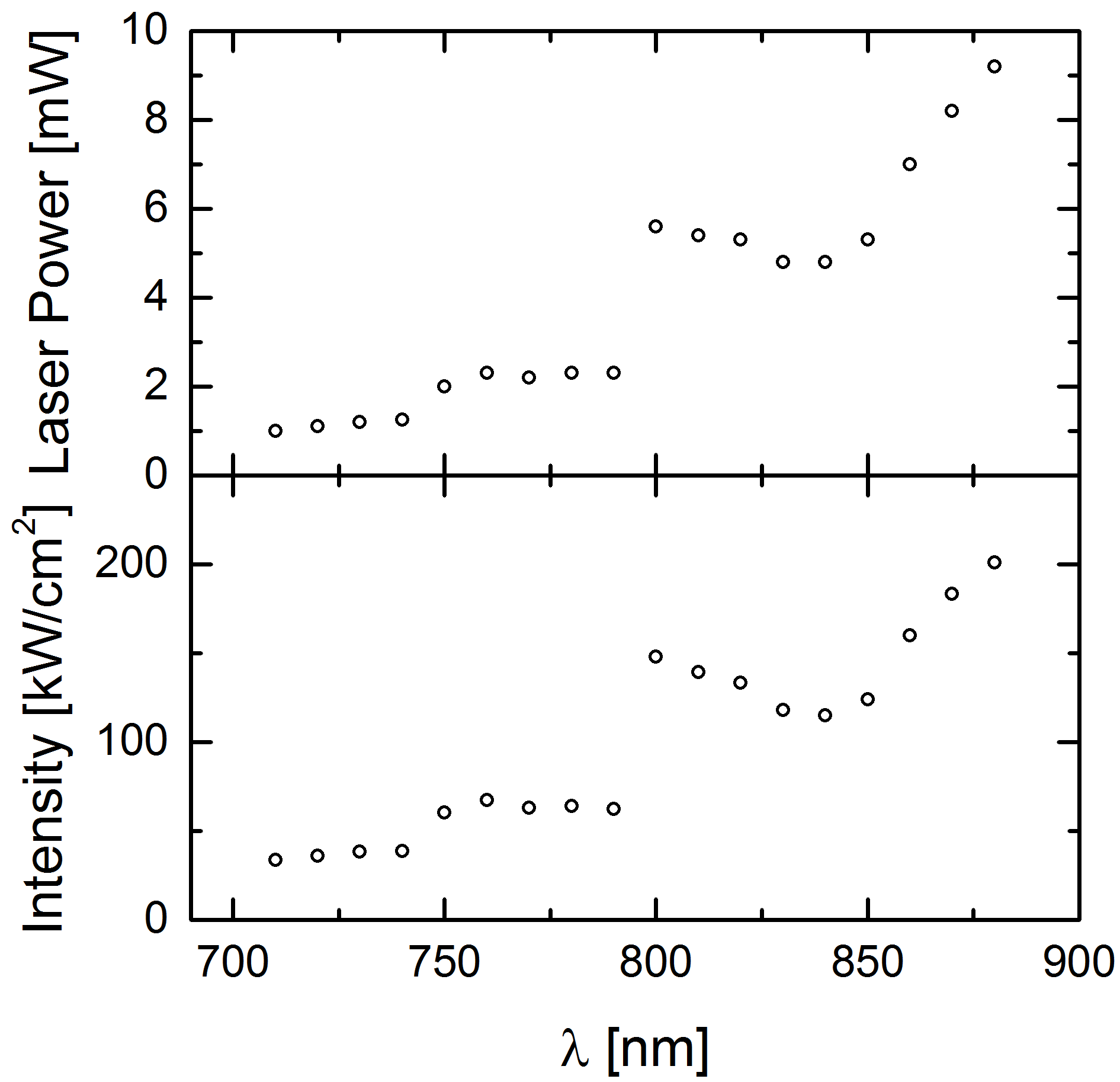


Figure S3: Top) Laser power of the Ti:sapphire laser as a function of excitation wavelength. The power was obtained by measuring the laser power directly in front of the microscopy beam entrance. Bottom) Laser intensity as obtained from the laser power by taking into account the 1/e^2^ – area of the point spread function of the focused laser spot and losses due to beam splitter and objective.
